# Meetings

**Published:** 1891-03

**Authors:** 


### Bristol Medico-Chirurgical Society.

*January 14th*, 1891.

Mr. S. H. SWAYNE, President, in the Chair.

Dr. F. H. EDGEWORTH exhibited specimens and microscopic sections from cases of cystic disease of the liver and malignant disease of the pylorus. In the former, which was from a female patient who died from peritonitis following herniotomy, all the bile passages were dilated, the common duct admitting a lead-pencil; the gall bladder was distended and contained biliverdin granules. Cysts, varying in size from minute specks to two inches in diameter, were scattered throughout the liver. These were full of a white opalescent fluid and lined by pavement epithelium. The condition was probably due to retention, not to primary cystic disease of the organ.

In the second case there was a nodular ulcerating growth at the pyloric end of the stomach, with secondary growths in the liver and great omentum. The left ovary was uniformly enlarged by a deposit, which under the microscope showed the features of an adenoma, as did sections of the other affected parts. It was thought that the ovary was involved by the transference of particles through stomata in the epithelium at the hilus of this organ.

Mr. C. A. Morton showed (1) a case of branchial fistula in a child aged 10, with a retention cavity behind the opening; (2) a girl aged 10, with uni-lateral congenital dislocation of the hip; and (3) microscopic sections of a sarcoma of the thigh showing small cells and branching cells forming a network with smaller ones in the meshes.

Dr. J. E. Shaw exhibited a heart from a woman aged 38, who had suffered from congenital cyanosis. The organ had the following peculiar abnormalities: The aorta sprang from the right ventricle, the pulmonary artery from the left, and the two vessels were connected by a large and pervious ductus arteriosus. There was a patent foramen ovale, which would admit the tip of a finger; and in the upper part of the intra-ventricular septum was an opening one inch in diameter. The coronary vessels were extremely large and tortuous, being more than half an inch in diameter at their origin from the aorta.

Mr. Munro Smith exhibited microscopic sections from three cases of sarcoma of the tibia, which illustrated the various modes of growth of these tumours, and showed a patient who was affected with scirrhus of both breasts and secondary nodules scattered over the thorax. There were also deposits in both eyelids and orbits.

Dr. Michell Clarke showed a patient who had suffered from locomotor ataxy for some years, in whom the knee-jerk was still present.

Mr. L. M. Griffiths exhibited a placenta which had been firmly adherent, and which he had removed that morning. There were extensive deposits of white, firm material on the surface.

---

*February 11th*, 1891.

Mr. S. H. Swayne, President, in the Chair.

Dr. G. Parker showed a patient who had suffered during the last 18 months from several attacks of transient left hemiplegia, each attack being followed by copious hæmaturia. The urine was normal between the seizures. Choreic movements of the left hand had often been present at the commencement, and had now become permanent. Consciousness was never lost. There was an old history of hæmaturia. The symptoms possibly depended on simultaneous hemorrhage from degenerated arteries in the brain and kidney.

Mr. W. R. ACKLAND read a short paper on the care of the teeth in childhood and adult life. He pointed out that the ordinary causes of decay were acid solvents in the saliva (due to gout, rheumatism, indigestion, &c.) and germs. Acid and ferruginous medicines, sugar, smoking, and heredity were also discussed as possible factors. Dr. Scanes Spicer's paper on the influence of mouth-breathing was mentioned. Hints as to the preservation of the teeth by alkaline lotions, antiseptic powders, the tooth-brush, &c., were given. The consequences of overcrowding and consequent irregularity of the teeth, loosening due to mercury, periostitis, &c., were touched upon, and finally some practical methods of relieving toothache were mentioned.

Dr. MICHELL CLARKE read notes on two cases of intra-cranial tumours, and showed specimens of the same in sections of the brains. The first was from a man who had suffered from weakness of the left leg, increasing to complete paralysis of the left side, with severe occipital headache, vomiting, and finally coma. The tumour was syphilitic, and situated below the island of Reil on the right side. The other case was a glioma in the left optic thalamus of a girl aged six years.

Dr. WATSON WILLIAMS read a paper on "The Therapeutics of Koch's Lymph." After briefly reviewing the physiological action of the fluid in tubercular and non-tubercular cases, he pointed out that the effects were probably due, not to chemical combination with the diseased tissue, but to stimulation of the defensive cells of the part, to successfully resist the invading bacilli. This was borne out by the facts that the amount of reaction was in proportion to the susceptibility of the patient, rather than to the amount of tubercular tissue, and that the best results sometimes occurred where there was least reaction. A comparison was drawn between this and the action of mercury in syphilis, which John Hunter considered to be due to stimulation of the tissues.

---

*March 11th*, 1891.

Mr. S. H. SWAYNE, President, in the Chair.

Mr. S. H. SWAYNE referred to the death of Dr. Frederick Brittan, the first President of the Society, and proposed that a letter of condolence and sympathy should be forwarded to his widow. This was seconded by Dr. Fox, and unanimously resolved.

Dr. WALDO exhibited two patients; viz., (1) A case of supposed lupus vulgaris in a girl which had been treated with Koch's lymph. There had been a well-marked general reaction, but no local one. (2) A boy with an eruption on his forehead of small flat papules, with shining surfaces resembling lichen planus.

Dr. HARRISON showed a well-marked case of molluscum fibrosum in a woman aged 47. The disease began when she was 14 years old.

Dr. SHINGLETON SMITH exhibited an interesting specimen of non-malignant stenosis of the pyloric orifice, from a female patient

6

aged 33. She had enjoyed fairly good health until October, 1889, when she had an attack of jaundice without obvious cause. This never quite cleared up. Vomiting came on, and gradually increasing emaciation. The vomited matter invariably contained bile. There was no hepatic enlargement, and no thickening of the pylorus to be felt. The patient was highly neurotic There was a small fibroid tumour connected with the uterus. Emaciation became extreme, and in spite of persistent rectal feeding she died from inanition. At the autopsy it was found that there was an extremely small pyloric orifice which admitted a No. 8 catheter, with no signs of morbid growth or muscular hypertrophy. There was no evidence of organic disease in other organs. The gall-bladder contained a quantity of bile. Dr. Smith considered this to be, probably, a congenital condition, and referred to Landerer's report of cases of this nature.

Mr. Pagan Lowe read notes on a case of pemphigus vegetans which occurred in a married woman aged 47, and resulted fatally three and a half months from the initial symptoms. The lips were everted, swollen, and covered with black crusts. Both eyelids were affected in the same way. The axillæ had a scalded appearance, and in the groins the epidermis was raised in thick, moist, leathery masses. Several pemphigus spots were dotted about the body and limbs. There was great pain in movement owing to the excoriations. Few cases of this remarkable disease are on record. Of these, only two (under Mr. Jonathan Hutchinson) recovered under treatment by opium.

G. Munro Smith, *Hon. Sec.*

